# An assessment of the prevalence of Aflatoxin M1 level in milk and milk powder based on high performance liquid chromatography and dietary risk assessment

**DOI:** 10.1016/j.toxrep.2024.101787

**Published:** 2024-11-09

**Authors:** Yaser Almasoud, Manar Alwelaie, Zeyad Aldosari, Sarah Alotaibi, Abdullah Alsayari, Ghada Alzyadi, Mohammed Almutairi, Sulaiman Alajel, Abdullah Al Tamim, Hibah Alharbi

**Affiliations:** Saudi Food and Drug Authority, Riyadh 0112038222, Saudi Arabia

**Keywords:** Risk, Health, M1, Milk, Aflatoxins, LC-MS/MS

## Abstract

Milk is susceptible to Aflatoxin M1 (AFM1) contamination, mainly through consumption of contaminated animal's feed. The natural occurrence of aflatoxin M1 (AFB1) has been surveyed in samples of milk and milk powder. Samples collected from different regions of Saudi Arabia during the period from 2013 to 2021 were subjected to high-performance liquid chromatography analysis, and (AFB1) was quantified with detection limits of 0.5 µg/kg. The Saudi Food and Drug Authority has taken strong actions to limit AFM1 contamination, increase aflatoxin control, and modify the regulations. A study analyzed 363 randomly collected milk samples (168 milk, 195 milk powder) from Saudi Arabia using LC-MS/MS. Out of the 363 samples, 20 were positive for AFM1, with 343 were within the limits. One out of the 168 milk samples and 19 out of the 195 milk powder samples exceeded the limits, with seven originating from Saudi Arabia. The highest AFM1 concentration (3.97 µg/kg) was found in the Saudi samples. Saudi Arabia's mycotoxin regulations significantly reduced AFM1 contamination in milk, which is not considered a serious health hazard.

## Highlights

• Examine aflatoxin M1 contamination in milk and its associations with significant health concerns.
• Regulatory modifications diminished aflatoxin M1 exposure in Saudi Arabia by 90 % following 2019.
• Analysis of 363 milk samples indicates that 5.5 % above acceptable aflatoxin M1 levels in Saudi Arabia.
• Risk evaluation indicates diminished carcinogenic risks resulting from revised aflatoxin M1 thresholds.
• A high-resolution LC-MS/MS technique has been developed for the detection of aflatoxin M1 in milk.
• Method accuracy validated with 98.5–110 % recovery and minimal detection limits.

## Graphical Abstract

**Figure:**
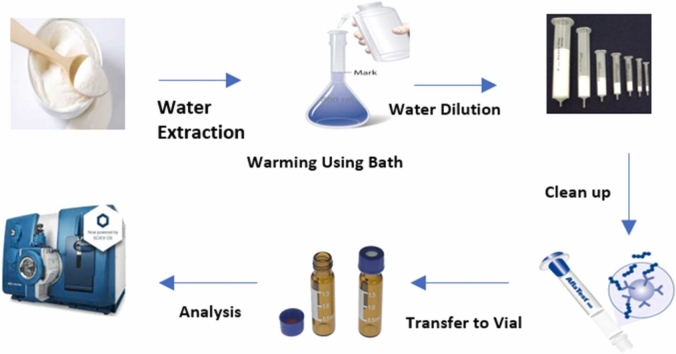

## 1 Introduction

Mycotoxins are low molecular weight natural products that are produced by filamentous fungus of the genera Fusarium, Penicillium, and Aspergillus on various agricultural crops [15]. The most frequent mycotoxin-producing fungus is known to be Aspergillus, which is responsible for manufacturing a variety of life-threatening mycotoxins, such as aflatoxins, ochratoxins, cyclopiazonic, aflatrem, terrein, gliotoxin, citrinin sterigmatocystin, and patulin [22], [31]. Depending on environmental parameters such as humidity, water activity, temperature, and agricultural techniques, fungal growth and mycotoxin emission can occur at any stage of crop production (cropping, harvesting, and storage) [40]. The optimal temperature for aflatoxins formation has been determined to be 30 °C. Unfortunately, because these toxins are stable at high temperatures and cannot be removed by heat treatment, removing contamination with heat during industrial food processing remains a challenge. As a result, these poisons can be discovered in infants’ food, children's food, and bakery items [4]. Crops that have been contaminated with mycotoxins pose a severe hazard to public health; hence, mycotoxins have piqued the interest of human, animal, and plant health professionals [32], [36]. The Food and Agriculture Organization (FAO) predicts that the current mycotoxin levels are 25 % higher than the Codex and European Union standards [16]. By establishing regulatory rules, the World Health Organization (WHO) has attempted to address the global problem of mycotoxin contamination in food chains. Furthermore, the joint scientific advisory committee declared that it is responsible for assessing the health concerns arising due by mycotoxins [10], [15], [39].

The most frequently reported mycotoxins are aflatoxins, which consist of four compounds: aflatoxins B1, B2, G1, and G2 [22]. Studies indicate that aflatoxins are commonly found in agricultural crops in warm climates, such as corn, grains, and peanuts. are commonly found in warm-climate agricultural crops, such as maize, grains, and peanuts [7]. The development of aflatoxins and the invasion of agricultural goods is a lengthy biochemical process that begins with fungus growth before progressing to toxin synthesis [2]. The biosynthesis of aflatoxin consists of 13 enzymatic steps that begin with the conversion of acetate to norsolorinic acid. Up to 30 genes are implicated in the fungal synthesis of aflatoxins [41]. The main fungi that produce aflatoxins are Aspergillus flavus and Aspergillus parasiticus and these toxins are not produced by all Aspergillus species, and not all Aspergillus species infest agricultural products. Therefore, the levels and severity of aflatoxin contamination of agricultural products are, to a certain extent, determined by the fungal ecology of the production field [13], [37]. Aflatoxins are toxic compounds and linked to various harmful health effects, including acute toxicosis at high dosages, immunological dysfunction, and impaired infant growth [8], [11], [20], [35]. Moreover, aflatoxins have been linked to liver cancer in both human and various animal species for almost 60 years. "Naturally occurring combinations of aflatoxins" have been classified by the International Agency for Research on Cancer (IARC) as a group 1 human carcinogen [19].

Milk is a healthy, wholesome dairy product, that is consumed by the majority of the world's population for drinking and also as an element of other dairy products [28]. The fact that it is easily and quickly absorbed makes it essential for newborns, nursing mothers, toddlers, and the elderly. As well as providing amino acids, milk proteins are also necessary for the development of both adults and newborns [3]. The contamination of milk and dairy products with different mycotoxins has been demonstrated worldwide as a result of mycotoxin’s presence in animal feed and the subsequent generation of contaminated milk [19], [25]. These commodities are primarily contaminated with aflatoxin M1, which represents the most important and also the most toxic metabolite of aflatoxin [12], [27]. The aflatoxin M1 contamination of milk results primarily from the conversion of aflatoxin B1, that is metabolized by enzymes found primarily in the liver. After aflatoxin M1 has been formed, it is excreted through the urine and milk of the cow [26]. Aflatoxin M1 causing acute hepatotoxicity was first observed in a study on ducklings, where the birds underwent oral exposure to aflatoxin M1 [9], [29]. Additionally, aflatoxin M1 has been demonstrated to have direct toxic potential in human cell lines, again without the need for metabolic activation [23]. Moreover, many in vivo research endeavors have shown that aflatoxin M1 suppresses both the innate and adaptive immune responses [34]. Consequently, milk consumers are at risk of a potentially high exposure to Aflatoxin M1. The European Union (EU) has set the maximum permissible limit for Aflatoxin M1 in milk at 0.5 µg/Kg. This low limit creates a need for an accurate, reliable method to detect and quantify Aflatoxin M1. The action level for aflatoxin B1 is 20 parts per billion for feed fed to lactating dairy cows. As both aflatoxins B1 and M1 may cause cancer in humans, the action level of 0.5 parts per billion of aflatoxin M1 in milk is strictly enforced by the United States Food and Drug Administration (FDA) [26]. Therefore, a simple method was developed and validated for determination of aflatoxin M1 in milk at proposed regulatory limits.

The tested sample were mixed with water, filtered, and applied to an immunoaffinity column. The column was washed with Phosphate buffered saline, and aflatoxin was eluted with mix of methanol, acetonitrile and water. Chromatographic separation was achieved on Kinetex C18 column (2.1 ×100 mm. 2.6 µ) coupled to 6500 triple quadruple mass analyzer. The Parameters used to validate the method include selectivity, linearity, precision, limit of detection (LOD), and quantification (LOQ). Precision was tested by spiking milk samples with 0.1 and 0.20 µg/kg aflatoxin M1. Recoveries ranged from 98.5 % to 110 %, and the method was found to be linear over a concentration of 0.1 – 2 ng/ml. The limit of detection and quantification were established at 0.011 and 0.014 µg/kg, respectively. This method gives high resolution, lower retention time, low LOQ. The evaporation step was eliminated, providing a rapid and accurate method for the determination of AFL M1 in milk.

Before these regulations were implemented, this appeared to be a public health problem, based on the results of our investigations. These inspections subsequently led to a new regulation by the Saudi authorities to control the presence of aflatoxin M1 in both milk and milk powder. Furthermore, this study will evaluate the estimated exposure and risk assessment regarding aflatoxin M1, which reflects the importance of controlling and preventing AFM1 in milk products.

## 2 Material and methods

### 2.1 Chemical and reagents

Aflatoxin M1 standard solution was purchased from Trilogy Analytical Laboratory (Trilogy®, USA). The Immunoaffinity column (IAC) was purchased from R-Biopharm (Germany) and the 150 mm filter paper was obtained from Whatman (USA). High performance liquid chromatograph (HPLC) grade acetonitrile (ACN) and HPLC grade methanol were obtained from Merck (Germany). HPLC grade water was obtained from Fisher (USA) and, finally, phosphate buffered saline (PBS) was purchased from Sigma Aldrich (USA).

### 2.2 Sampling

Three hundred and sixty-three (363) samples of milk and milk powder, that were randomly collected from different regions of Saudi Arabia during the period from 2013 to 2014 (n=77), were analyzed using liquid chromatography (LC) and high-performance liquid chromatography (HPLC). In addition, 286 samples of milk and milk powder, collected between 2015 and 2021, were analyzed using liquid chromatography-coupled quadrupole tandem mass spectrometry (LC–MS/MS).

### 2.3 Sample preparation

The milk powder was obtained from the local market and 10 g of each sample was weighed into a 250 ml glass beaker. The extraction protocol entailed adding 50 ml of HPLC water, then pre-warming in a water bath at 50 °C. The mixture was then homogenized by shaking, then allowed to cool to room temperature (20–25 °C), before being transferred into a 100 ml volumetric flask and diluted with water to achieve a final volume of 100 ml. It was then shaken for 15 minutes, and centrifuged at a radial acceleration of at least 5000 rpm for 15 min. The test solution was then filtered through 150 mm Whatman filter paper. Fifty (50) ml of the mixture was then passed through the immunoaffinity column (IAC) at a rate of 2–3 ml/min, which was followed by the washing step, using 20 ml of phosphate buffered saline (PBS). Slight pressure was applied on the top of the IAC to ensure that the column was completely dry after washing. As the next step, the Aflatoxin M1 was eluted using a 1.25 ml of Methanol: Acetonitrile (4:6), then 1.25 ml of HPLC water. The filtrate was collected, shaken, then transferred to an LC-MS/MS vial for analysis.

### 2.4 Standard solutions

The stock standard solution was prepared at a concentration of 10 ng/ml in can, before being stored below 8° C. On the analysis day, working standard solutions were prepared in 10 % acetonitrile at seven different concentrations (0.02, 0.05, 0.1, 0.2, 0.5, 1, and 2 ng/ml) for LC–MS/MS analysis. For the HPLC - fluorescence detector (FLD) analysis, the working standard solutions were prepared in 10 % acetonitrile at five different concentrations (0.1, 0.2, 0.5, 1, and 2 ng/ml of Aflatoxin M1 working standards from the stock standard 10 ng/ml, dilute using acetonitrile 10 % and stored in a freezer at a temperature from −18 to −20ºC.

### 2.5 Instrument

The LC-MS/MS system contained an Agilent 1290 system (Santa Clara, CA, USA), equipped with a Binary Solvent pump, an autosampler and a column heater with active pre-heating attached to a 6500 Qtrap mass analyzer (AB Sciex, Canada). Nitrogen gas was used as collision gas, curtain gas and nebulizer gas on the MS. The separation was performed using Phenomenex Kinetex CI8 (100 mm×2.1 mm; 2.6 µm) with a C18 guard column (Phenomenex, Torrance, CA, USA). For the autosampler, the Syringe Size was (20 µl), the Injection Volume (5 µl) and the left and right temperature of the column was 45 °C.

### 2.6 LC-MS/MS analysis

The mobile phase consisted of A (5 mM ammonium formate in HPLC grade water with 0.1 % formic acid) and B (5 mM Ammonium formate in 50/50 Acetonitrile/methanol with 0.1 % formic acid). The linear gradient elution was isocratic 40 % B. The flow rate was set at 0.3 ml/min, while the column temperature was set at 20 ºC and the injection volume was 5 µL. Separation was carried out on a Kinetex C18 column (2.1 ×100 mm. 2.6 µ) (phenomenex, USA), Attached to 1290 liquid chromatography system (Agilent, USA), coupled with a 6500 Qtrap mass analyzer (AB Sciex, Canada). Electrospray ionization mass spectra (ESI-MS) were acquired in the positive (ES +), and the Turbo Ion source operated at 500 ºC, with an electrode voltage at 5500 V. The other MS parameters were as follows: curtain gas, 30 psi; collision gas, 12 psi; and entrance potential, 10 V.

## 3 Results and discussion

**Fig. 1:**
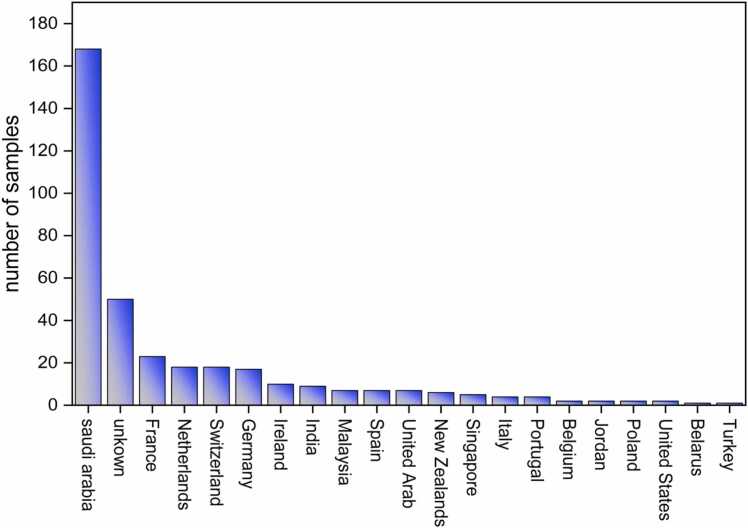
Country of origin of the samples used in the study.

**Fig. 2:**
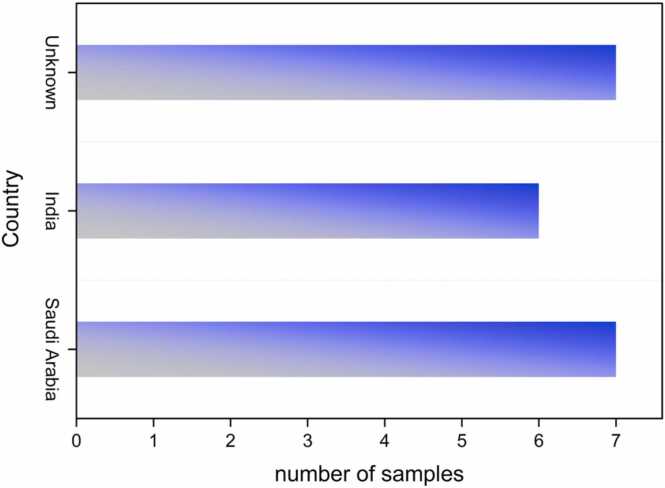
Countries where the limit for Aflatoxin M1 is exceeded.

The milk and milk powder samples from different countries were analyzed using LC-MSMS for the period from 2013 to 2022 (Fig. 1). Out of the total of 363 analyzed samples, twenty were positive for Aflatoxin M1 (AFM1) (5.50 %) and 343 were within the limit (94.5 %). Nineteen (19) out of the 195 milk powder samples exceeded the limit (10 %), while seven out of 168 milk samples (4.2 %) that originated from Saudi Arabia were contaminated with AFM1. Additionally, five out of the nine milk powder samples from India were contaminated with AFM1 (62.5 %), and seven out of 57 unknown samples (12 %) tested positive for AFM1. The highest concentration of AFM1 was found in the samples from Saudi Arabia, at 3.97 µg/kg. One out of the 168 milk samples (0.6 %) that exceeded the limit for Aflatoxin M1 was from India, with a concentration of 0.56 µg/kg (Fig. 2), whereas, the maximum limit laid down by the Saudi Food and Drug Authority (SFDA) for aflatoxin M1 in milk and milk powder is 0.5 µg/kg (Table 1). In our research, we calculated the exposure assessment of Aflatoxin M1 in the milk samples in two stages: before the modifying regulation was introduced in 2019 and after the SFDA regulation was amended over the past three years (2020, 2021, 2022). According to our results, the exposure to aflatoxin through milk consumption in Saudi Arabia ranged from 0.0015 to 0.0037 μg/kg-b-w/day before the regulation was modified. Following the modification of the SFDA regulation, exposure to Aflatoxin M1 decreased to between 0.0001 and 0.0003 μg/kg-b-w/day, indicating a lower exposure level. The results for the margin of exposure (MOE) and hazard quotient (HQ) indicated the risk of both a carcinogenic and non-carcinogenic effect prior to 2019 while, after 2019, there has been a clear improvement in the health risk assessment indicators due to the control of Aflatoxin M1 in Saudi Arabia, which has played an important role in reducing the contamination of milk products by Aflatoxin M1.

**Table 1:** Occurrence and Frequency of Aflatoxin M1 Levels in Milk and Milk Powder Samples.

**Type of Samples**	**No. of Samples**	**Samples Exceeding SFDA ML**	**% Samples Above SFDA ML**	**Within Limit**	**% Within Limit**	**Exceeded Range (µg/kg)**	**SFDA ML (µg/kg)**
**Milk Powder**	195	19	10.0 %	176	90 %	0.55–3.97	0.5
**Milk**	168	1	0.6 %	167	99.4 %	0.56	0.5
**Grand Total**	**363**	**20**	**5.5 %**	**343**	**94.5 %**	**0.55–3.97**	**0.5**

Aflatoxin M1 has been found in milk and milk powder, with different levels of contamination in many countries, including Gulf Cooperation Council (GCC) as well as other Middle Eastern countries. Previous research conducted in Najran City, Saudi Arabia, found that all of the positive samples were below the acceptable level for AFM1. Of the 96 milk samples analyzed, 82 % were contaminated with aflatoxin M1 in concentrations that varied from 10 to 190 ng/L. It can be concluded that AFM1 contamination in dairy products in Najran city does not appear to pose a serious public health problem at present Medhat I.M. Abdallah [1] while, in Jeddah City, 58 % of the milk samples in Saudi Arabia were positive for aflatoxin M1 [6]. Other research conducted in Saudi Arabia using 250 milk samples reported that 30 % of these were contaminated with aflatoxin M1 [5]. In Qatar, 73 % of the milk samples (milk, yogurt, cheese, butter and laban) were contaminated with aflatoxin M1, with concentrations ranging from 5 to 217 ng/L for all 96 of the milk samples analyzed [18]. In Kuwait, 361 samples of milk, breast milk and cheese were analyzed. Aflatoxin M1 was detected in 5 % of the milk samples, 41 % of the human milk samples and 79 % of the cheese samples, ranging from 5 to 88.8 ng/kg [14]. In Yemen, 254 samples of four types of milk products were analyzed. Aflatoxin M1 was detected in 68 % of the milk samples, 66 % of the powdered milk samples, 87 % of the yogurt samples, and 81 % of the cheese samples, ranging from 21 ng/L to 5950 ng/L [21]. Dairy products used in Yemen showed an AFM1 content that exceeded the acceptable limit. This is a major factor in causing health-related complications, including cancer. A total of 175 milk samples were collected in Jordan for the period from 2014 to 2015. All of the tested samples were contaminated with various levels of AFM1, ranging from 9.71 to 288.68 ng/kg [24] (Table 2).

**Table 2:** Occurrence of Aflatoxin M1 levels in milk and milk products from different origins.

Country	Sample type	Range of sampling results	Used regulation	Ref.
Saudi Arabia- Najran	milk	10–190 ng/L	-----	[1]
Qatar	Milk, yogurt, cheese, butter and Laban	5–217 ng/L	European Union	[18]
Kuwait	Milk, powdered milk, human milk, cheese	5–88.8 ng/kg	-----	[14]
Yemen	Milk, powdered milk, yogurt, cheese	21–5950 ng/L	European Union	[21]
Saudi Arabia- Jeddah	Milk	-----	European Union	[6]
Saudi Arabia	Milk	-----	SASO	([5])
Jordan	Milk	9.71–28.7 ng/kg	-----	[24]

## 4 Risk assessment of Aflatoxin M1

Milk and milk products are commonly consumed on a daily basis all over the world and form an especially important aspect of children's diets, who may be more sensitive to adverse effects from aflatoxin M1 [17]. Regarding the presence of aflatoxin M1 in milk and other dairy products, numerous nations across the world have established regulations pertaining to food and consumer safety. For that reason, the SFDA have sought to promote consumers’ safety in Saudi Arabia by implementing regulations that are designed primarily to protect the Saudi market from contaminated food products. Under the SFDA regulation that was in operation prior to 2019, we found that 25 % of the milk samples exceeded the limit set by the SFDA. The increasing control of aflatoxin and modifications of the regulation mean that, by 2022, none of the samples exceeded the limit.

Methods: The process of determining the amounts of a chemical (or microbe, or other harmful agent) to which an individual should be exposed is known as an exposure assessment. In the case of dietary chemical exposure to aflatoxin M1, this process is frequently measured in terms of milligrams or micrograms per kilogram of bodyweight per day. Our exposure assessment determined how much milk powder adult and children populations consume across Saudi. Hence, this could be extrapolated in order to an calculate the average daily dose (ADD), as follows:

(1) $$\mathrm{ADD}=\;=\;\frac{C\times \mathrm{CR}}{\mathrm{BW}}$$

For a risk characterization of aflatoxin M1 with regard to different non-carcinogenic effects, we calculated the Hazard Quotient (HQ):

(2) HQ = ADD/RfD (0.0002 μg/kg/day)

For the carcinogenic effect, we employed the margin of exposure (MOE):

(3) MOE = Benchmark dose (lower confidence limit), BMDL10 (0.57 μg/kg/day)/ADD

(BMDL_10_): the benchmark dose lower confidence limit

This study illustrates the exposure assessment of Aflatoxin M1 in milk samples that were indicated as outside the limit using two stages: before the SFDA regulations were modified in 2019 and after they were amended in the subsequent three years (2020, 2021, 2022), to compare the results for the exposure and risk assessment before and after the SFDA regulations were introduced. Table 3 shows the results for the exposure and HQ & MOE value of aflatoxin M1 in adults and children in Saudi Arabia. According to our findings, the exposure to aflatoxin through milk consumption in Saudi Arabia was 0.0015–0.0037 μg/kg-b-w/day before the regulations were modified. After the SFDA regulations were modified, the exposure to aflatoxin M1 decreased to approximately 0.0001–0.0003 μg/kg-b-w/day), which indicates a lower exposure level compared to other countries (Brazil, Ethiopia, and Mexico). Additionally, our results indicated a risk of both carcinogenic and non-carcinogenic effects before 2019, while, after 2019, the carcinogenic risk decreased, and there was no indication of any non-carcinogenic risks due to aflatoxin in the milk samples after the regulations were modified. Table 4 shows previous studies in which dietary exposure to aflatoxin was calculated, so that we may compare their results with those of our study. Since no country or international standard-setting organization (such JECFA) has yet established a tolerable daily intake for aflatoxin M1, we are unable to decide whether the exposure in various world populations has an adverse effect on human health or not. Nonetheless, based on the available information about adverse, aflatoxin M1-induced health effects from in vivo and in vitro toxicological studies, it appears advisable that the exposure to aflatoxin M1 should be kept as low as reasonably achievable. For this reason, after SFDA implemented the regulations, the concentration and exposure to aflatoxin M1 decreased to zero, making the milk products that are available on the Saudi market safer for consumption by all age groups.

**Table 3:** The results for the exposure and HQ & MOE value of aflatoxin M1 for the two age categories (adults and children) in Saudi Arabia.

	Saudi Population	ADD (Μg/Kg-B-W/Day)	MOE	HQ
Before 2019	Adult	0.00159	137.27	7.95
	Children	0.0037	70.703	11.2
After 2019	Adult	0.0001	3211.196	0.5
	Children	0.0003	1449.558	1.5

**Table 4:** Previous studies in which dietary exposure to aflatoxin was calculated, to facilitate a comparison of our results with the results of our study.

Country	Mean Conc. Of AFM1	ADD (μg/kg-b-w/day)	Ref.
Brazil	1.5	0.6934	[33]
Ethiopia	0.97	0.7891	[38]
Mexico	0.495	2.3056–2.638	[30]
Present study	1.385	0.0001–0.003	---

## 5 Conclusion

In this study, we undertook an exposure assessment of Aflatoxin M1 in milk samples that were indicated as being above the limit. We observed two stages: before the regulations were modified in 2019 and after the SFDA regulations have been amended over the three subsequent years (2020, 2021, 2022), in order to compare the results related to exposure and risk assessment before and after the SFDA regulations were implemented, to demonstrate the result of the exposure and HQ & MOE value of aflatoxin M1 in adults and children in Saudi Arabia. The control of mycotoxins in Saudi Arabia has played an important role in reducing the aflatoxin M1 contamination of milk, and this is not considered a serious threat to human health. The risk level is low, and >94.5 % of the samples were within the limit (>5.5 % of aflatoxin M1). Our results were found to be similar to those reported by many related studies from around the world.

## Author statement

We confirm that the manuscript has been read and approved by all named authors and that there are no other persons who satisfied the criteria for authorship but are not listed. We further confirm that the order of authors listed in the manuscript has been approved by all of us.

## CRediT authorship contribution statement

**Mohammed Almutairi:** Writing – review & editing, Validation, Conceptualization. **Ghada Alzyadi:** Writing – original draft, Formal analysis, Data curation. **Sulaiman Alajel:** Writing – review & editing, Conceptualization. **Yaser Almasoud:** Writing – original draft, Validation, Supervision, Software, Methodology, Formal analysis. **Hibah Alharbi:** Writing – review & editing, Conceptualization. **Abdullah Al Tamim:** Writing – review & editing, Validation, Conceptualization. **Abdullah Al Sayari:** Validation, Supervision, Conceptualization. **Sarah Alotaibi:** Writing – original draft, Methodology, Formal analysis. **Zyad Aldosari:** Validation, Formal analysis, Data curation, Conceptualization. **Manar AL Welaie:** Writing – original draft, Validation, Methodology, Investigation, Formal analysis.

## Declaration of Competing Interest

The authors declare that they have no known competing financial interests or personal relationships that could have appeared to influence the work reported in this paper.

## Data Availability

Data will be made available on request.

## References

[1] Abdallah M.I.M., Bazalou M.S., Al-Julaifi M.Z. (2012). Determination of aflatoxin M1 concentrations in full-fat cow’s UHT milk sold for consumption in Najran-Saudi regarding its public health significance. Egypt. J. Appl. Sci..

[2] Abrar M., Anjum F.M., Butt M.S., Pasha I., Randhawa M.A., Saeed F., Waqas K. (2013). Aflatoxins: biosynthesis, occurrence, toxicity, and remedies. Crit. Rev. Food Sci. Nutr..

[3] Afzal A., Mahmood M.S., Hussain I., Akhtar M. (2011). Adulteration and microbiological quality of milk (a review). Pak. J. Nutr..

[4] Ahlberg S., Randolph D., Okoth S., Lindahl J. (2019). Aflatoxin binders in foods for human consumption—can this be promoted safely and ethically?. Toxins.

[5] Al-Hizab F.A., Barakat S.E.M., Hussein Y.A. (2022). Occurrence of Aflatoxins in livers and milk of camels in Saudi Arabia. Vet. Med. Public Health J..

[6] Al-Kenani, A.A. (2014). Detection of Aflatoxin M1 in Camel Milk in Jeddah Province.

[7] Alshannaq A.F., Gibbons J.G., Lee M.-K., Han K.-H., Hong S.-B., Yu J.-H. (2018). Controlling aflatoxin contamination and propagation of Aspergillus flavus by a soy-fermenting Aspergillus oryzae strain. Sci. Rep..

[8] Azziz-Baumgartner E., Lindblade K., Gieseker K., Rogers H.S., Kieszak S., Njapau H., Schleicher R., McCoy L.F., Misore A., DeCock K. (2005). Case–control study of an acute aflatoxicosis outbreak, Kenya, 2004. Environ. Health Perspect..

[9] Ben Salah-Abbes J., Abbès S., Jebali R., Haous Z., Oueslati R. (2015). Potential preventive role of lactic acid bacteria against Aflatoxin M1 immunotoxicity and genotoxicity in mice. J. Immunotoxicol..

[10] Bennett, J., & Klich, M. (2003). chotoxins. C lin. Microbiol. Rev, 16, 497–516. 10.1128/CMR.16.3.497-516.2003 PMC164220 12857779

[11] Bondy G.S., Pestka J.J. (2000). Immunomodulation by fungal toxins. J. Toxicol. Environ. Health Part B: Crit. Rev..

[12] Cavret S., Lecoeur S. (2006). Fusariotoxin transfer in animal. Food Chem. Toxicol..

[13] Cotty P.J., Mellon J.E. (2006). Ecology of aflatoxin producing fungi and biocontrol of aflatoxin contamination. Mycotoxin Res..

[14] Dashti B., Al-Hamli S., Alomirah H., Al-Zenki S., Abbas A.B., Sawaya W. (2009). Levels of aflatoxin M1 in milk, cheese consumed in Kuwait and occurrence of total aflatoxin in local and imported animal feed. Food Control.

[15] El-Sayed R.A., Jebur A.B., Kang W., El-Esawi M.A., El-Demerdash F.M. (2022). An overview on the major mycotoxins in food products: characteristics, toxicity, and analysis. J. Future Foods.

[16] Eskola M., Kos G., Elliott C.T., Hajšlová J., Mayar S., Krska R. (2020). Worldwide contamination of food-crops with mycotoxins: validity of the widely cited ‘FAO estimate’of 25%. Crit. Rev. Food Sci. Nutr..

[17] Galvano F., Galofaro V., Galvano G. (1996). Occurrence and stability of aflatoxin M1 in milk and milk products: a worldwide review. J. Food Prot..

[18] Hassan Z.U., Al-Thani R., Atia F.A., Almeer S., Balmas V., Migheli Q., Jaoua S. (2018). Evidence of low levels of aflatoxin M1 in milk and dairy products marketed in Qatar. Food Control.

[19] Humans, I.W. G. on the E. of C.R. to, Cancer, I.A. for R. on, & Organization, W.H. (2002). Some traditional herbal medicines, some mycotoxins, naphthalene and styrene (Vol. 82). World Health Organization. PMC4781602 12687954

[20] In, I. (2015). Wild C.P., Miller J.D., Groopman J.D., editors. Mycotoxin Control in Lowand Middle-Income Countries. Lyon: WHO. 27030861

[21] Murshed S. (2020). Evaluation and assessment of aflatoxin M1 in milk and milk products in Yemen using high-performance liquid chromatography. J. Food Qual..

[22] Navale V., Vamkudoth K.R., Ajmera S., Dhuri V. (2021). Aspergillus derived mycotoxins in food and the environment: prevalence, detection, and toxicity. Toxicol. Rep..

[23] Nile S.H., Park S.W., Khobragade C.N. (2016). Occurrence and analysis of aflatoxin M1 in milk produced by Indian dairy species. Food Agric. Immunol..

[24] Omar S.S. (2016). Aflatoxin M1 levels in raw milk, pasteurised milk and infant formula.. Ital. J. Food Saf..

[25] Organization W.H., Cancer I.A. for R. on (1993). Some naturally occurring substances: food items and constituents, heterocyclic aromatic amines and mycotoxins. IARC Monogr. Eval. Carcinog. Risk Chem. Hum..

[26] Pennington, J.A. (2009). *Aflatoxin M1 in milk*. Cooperative Extension Service, University of Arkansas Division of ….

[27] Pleadin J., Frece J., Markov K. (2019). Mycotoxins in food and feed. Adv. Food Nutr. Res..

[28] Poonia A., Jha A., Sharma R., Singh H.B., Rai A.K., Sharma N. (2017). Detection of adulteration in milk: a review. Int. J. Dairy Technol..

[29] Purchase I.F.H. (1967). Acute toxicity of aflatoxins M1 and M2 in one-day-old ducklings. Food Cosmet. Toxicol..

[30] Quevedo-Garza P.A., Amador-Espejo G.G., Salas-García R., Ramos-Peña E.G., Trujillo A.-J. (2020). Aflatoxin M1 determination in infant formulae distributed in Monterrey, Mexico. Toxins.

[31] Ráduly Z., Szabó L., Madar A., Pócsi I., Csernoch L. (2020). Toxicological and medical aspects of Aspergillus-derived mycotoxins entering the feed and food chain. Front. Microbiol..

[32] Rao V.K., Shilpa P., Girisham S., Reddy S.M. (2011). Incidence of mycotoxigenic penicillia in feeds of Andhra Pradesh, India. Int. J. Biotechnol. Mol. Biol. Res..

[33] Scaglioni P.T., Becker-Algeri T., Drunkler D., Badiale-Furlong E. (2014). Aflatoxin B1 and M1 in milk. Anal. Chim. Acta.

[34] Shirani K., Riahi Zanjani B., Mehri S., Razavi-Azarkhiavi K., Badiee A., Hayes A.W., Giesy J.P., Karimi G. (2021). miR-155 influences cell-mediated immunity in Balb/c mice treated with aflatoxin M1. Drug Chem. Toxicol..

[35] Smith L.E., Prendergast A.J., Turner P.C., Humphrey J.H., Stoltzfus R.J. (2017). Aflatoxin exposure during pregnancy, maternal anemia, and adverse birth outcomes. Am. J. Trop. Med. Hyg..

[36] Soares R.R.G., Ricelli A., Fanelli C., Caputo D., de Cesare G., Chu V., Aires-Barros M.R., Conde J.P. (2018). Advances, challenges and opportunities for point-of-need screening of mycotoxins in foods and feeds. Analyst.

[37] Sohrabi N., Taghizadeh M. (2018). Molecular identification of aflatoxigenic Aspergillus species in feedstuff samples. Curr. Med. Mycol..

[38] Tadesse S., Berhanu T., Woldegiorgis A.Z. (2020). Aflatoxin M1 in milk and milk products marketed by local and industrial producers in Bishoftu town of Ethiopia. Food Control.

[39] Van Egmond H.P., Jonker M.A. (2004).

[40] Waliyar F., Osiru M., Ntare B.R., Kumar K.V.K., Sudini H., Traore A., Diarra B. (2015). Post-harvest management of aflatoxin contamination in groundnut. World Mycotoxin J..

[41] Yu J. (2012). Current understanding on aflatoxin biosynthesis and future perspective in reducing aflatoxin contamination. Toxins.

