# Supplementary material for: An assessment of the prevalence of Aflatoxin M1 level in milk and milk powder based on high performance liquid chromatography and dietary risk assessment
Source: Toxicol Rep. 2024 Nov 9;13:101787. doi: 10.1016/j.toxrep.2024.101787 (PMC11609700; doi:10.1016/j.toxrep.2024.101787)
Supplement: Supplementary file 1 — Supplementary material [file mmc1.docx]

**Supplementary**

| Analyte | Q1 Mass (Da) | Q3 Mass (Da) | Dwell (msec) | DP | EP | CE | CXP |
| --- | --- | --- | --- | --- | --- | --- | --- |
| Afla M1-I | 329.100 | 229.00 | 3.57 | 143 | 10.00 | 45 | 19 |
| Afla M1-II | 329.100 | 273.100 | 3.57 | 143 | 10.00 | 21 | 15 |

Table1: Tandem mass spectrometry acquisition parameters for Aflatoxin M1

| Analyte | Linear range (ng/mL) | Linear equation | Correlation Coefficient  ( r2) |
| --- | --- | --- | --- |
| Aflatoxin M1 | 0.05 - 2 | y = 1.53308e5 x + -422.46186 | 0.99991 |

Table2: The linearity of calibration curves


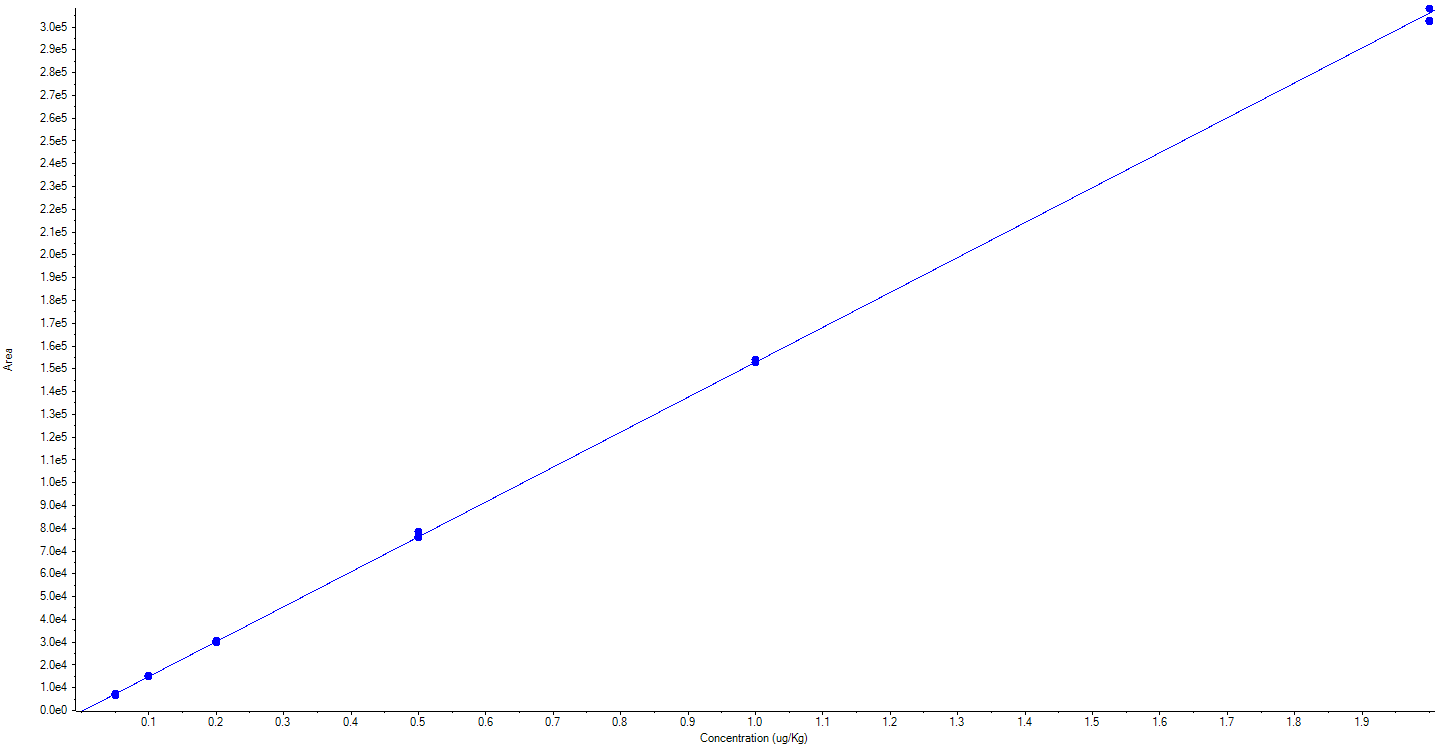


Figure1 Calibration curve, a correlation coefficient of (R2) > 0.999:

Figure2: Sample without spike and sample with spike of aflatoxin M1 " Sample ID: "" File: "20170131-YM.wiff- I" Mass(es): "329.100/273.100 Da"
